# Insight into the Neuroprotective Effect of Genistein-3′-Sodium Sulfonate Against Neonatal Hypoxic-Ischaemic Brain Injury in Rats by Bioinformatics

**DOI:** 10.1007/s12035-022-03123-8

**Published:** 2022-11-12

**Authors:** Ting Xie, Liyan Shuang, Gaigai Liu, Shanshan Zhao, Zhidong Yuan, Hao Cai, Lixia Jiang, Zhihua Huang

**Affiliations:** 1 Key Laboratory of Prevention and Treatment of Cardiovascular and Cerebrovascular Diseases of Ministry of Education, Gannan Medical University, Ganzhou, 341000 China; 2 Graduate School, Gannan Medical University, Ganzhou, 341000 Jiangxi China; 3 First Affiliated Hospital, Gannan Medical University, Ganzhou, 341000 China; 4 Basic Medicine School, Gannan Medical University, Ganzhou, 341000 China; 5 Pain Medicine Research Institute, Gannan Medical University, Ganzhou, 341000 China

**Keywords:** Genistein-3′-sodium sulfonate, Neonatal hypoxic-ischaemic encephalopathy, RNA-Seq, Bioinformatics, Neuroprotective

## Abstract

Therapeutic hypothermia (TH) is the only intervention approved for the treatment of neonatal hypoxic-ischaemic encephalopathy (HIE), but its treatment window is narrow (within 6 h after birth), and its efficacy is not ideal. Thus, alternative treatments are urgently needed. Our previous studies showed that genistein-3′-sodium sulfonate (GSS), a derivative of genistein (Gen), has a strong neuroprotective effect in rats with ischaemic stroke, but its role in HIE is unclear. A hypoxia–ischaemia (HI) brain injury model was established in neonatal male Sprague‒Dawley (SD) rats. Twenty-four hours after reperfusion, rats treated with GSS were assessed for cerebral infarction, neurological function, and neuronal damage. RNA-Seq and bioinformatics analysis were used to explore differentially expressed genes (DEGs) and regulated signalling pathways, which were subsequently validated by Western blotting and immunofluorescence. In this study, we found that GSS not only significantly reduced the size of brain infarcts and alleviated nerve damage in rats with HIE but also inhibited neuronal loss and degeneration in neonatal rats with HIE. A total of 2170 DEGs, of which 1102 were upregulated and 1068 were downregulated, were identified in the GSS group compared with the HI group. In an analysis based on Kyoto Encyclopedia of Genes and Genomes (KEGG) categories, the downregulated DEGs were significantly enriched in the pathways “Phagosome”, “NF-κB signalling”, and “Complement and coagulation cascades”, amongst others. Meanwhile, the upregulated DEGs were significantly enriched in the pathways “Neurodegeneration”, “Glutamatergic synapse”, and “Calcium signalling pathway”, amongst others. These results indicate that GSS intervenes in the process of HIE-induced brain injury by participating in multiple pathways, which suggests potential candidate drugs for the treatment of HIE.

**Graphical Abstract:**

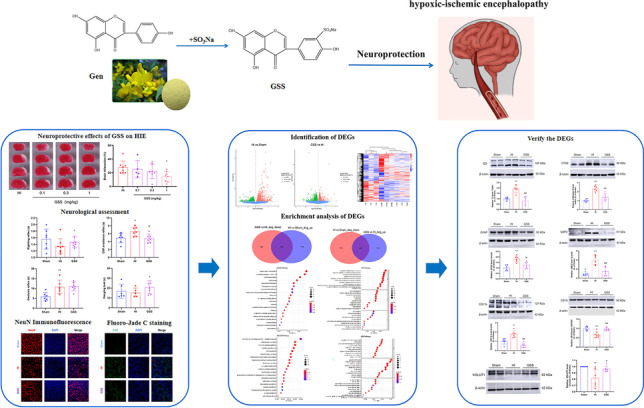

## Introduction

Neonatal hypoxic-ischaemic encephalopathy (HIE) is a disease in which partial or complete cessation of cerebral blood flow caused by ischaemia and hypoxia eventually leads to brain damage. HIE is the main cause of neonatal morbidity and mortality. According to reports, the incidence of neonatal HIE is approximately 1.5 per 1000 live births in developed countries and approximately 10–20 per 1000 live births in low- and middle-income countries [1]. In addition to ischaemia and hypoxia, physiological, cellular, and molecular changes in the brain lead to the development of various diseases, including cerebral palsy, epilepsy, disturbance of consciousness, intellectual disability, behavioural disturbances, and even neonatal death [2]. Recent studies have found that the mechanism by which HIE exerts detrimental effects is very complex and involves oxidative stress, intracellular Ca^2+^ accumulation, mitochondrial dysfunction, excitotoxicity, and inflammation [3]. At present, therapeutic hypothermia (TH) is the only clinically approved method for the treatment of HIE, but this treatment is ineffective in many children [4]. Additionally, strategies for the prevention and treatment of HIE in clinical settings are very limited [5]. Therefore, it would be of great significance to find effective drugs for the treatment of HIE.

Oestrogen is a cholesterol-derived steroidal hormone that acts in conjunction with its corresponding oestrogen receptor. Many studies have shown that oestrogen has a neuroprotective and therapeutic effect in a variety of neurological diseases [6], but long-term oestrogen therapy can increase the risk of breast cancer [7]. Therefore, phytoestrogens are used as an alternative to oestrogen. One such compound is genistein (Gen), an isoflavone compound extracted from legumes such as soybeans, clover, *Pueraria*, carob, and broad bean roots. Its chemical structure is similar to that of endogenous oestrogen [8], and it can bind to intracellular and cell surface membrane-bound oestrogen receptors to exert biological effects, such as antitumour, anti-inflammatory, antioxidant, and antiproliferative actions [9]. It has preventive and therapeutic effects in cancer, cardiovascular diseases, osteoporosis, diabetes, skin diseases, and other diseases [10]. Studies have shown that Gen has a neuroprotective effect; for example, it can protect rat hippocampal neurons from damage after cerebral ischaemia [11]. Although Gen has an oestrogen-like effect and causes fewer adverse reactions than oestrogen, its water solubility is poor [12]. Therefore, we sulfonated Gen to obtain a structurally modified product, genistein-3′-sodium sulfonate (GSS), which is characterised by strong water solubility and high bioavailability. Our previous research showed that GSS exerted a strong neuroprotective effect, not only significantly reducing cerebral infarct size and neurological function scores in rats with transient middle cerebral artery occlusion (tMCAO) but also protecting rat cortical neurons from glutamate-induced damage by inhibiting cell apoptosis [13]. Furthermore, GSS can upregulate α7nAChR expression to inhibit the NF-κB pathway, decrease the number of M1 microglia, and inhibit neuroinflammation in tMCAO rats [14, 15]. In this study, we found that GSS significantly reduced the cerebral infarct volume of neonatal HIE rats, which led us to suspect that it is a promising pharmacological agent to treat HIE, but its molecular target is still unknown.

The transcriptome refers to the sum of all RNA transcribed in a specific tissue or cell at a certain time or in a certain state, mainly including mRNA and noncoding RNA. RNA-Seq was developed more than 10 years ago and is an important tool in molecular biology. It reveals the degree of mRNA splicing and the regulation of gene expression by noncoding RNA and enhancer RNA, shaping our understanding of all aspects of genome function [16]. RNA-Seq has higher accuracy and sensitivity than traditional gene chips [17], which also makes it conducive to the discovery of new genes and new transcripts [18]. The current research shows that RNA-Seq has become a powerful method for studying the mechanisms of neurodegenerative diseases such as Alzheimer’s disease, Parkinson’s disease, and Huntington’s disease and discovering biomarkers for their diagnosis [19–22]. RNA-Seq is undisputedly an effective method to promote the discovery of new pathological mechanisms and therapeutic targets. Therefore, we used RNA-Seq technology to study GSS-induced changes in gene expression in neonatal rats with HIE and systematically identify the mechanism of action and therapeutic targets of the drug.

## Materials and Methods

### Drugs

GSS (C_15_H_10_O_8_SNa) was purchased from Shanghai Xishi Chemical Co., Ltd., diluted in normal saline to concentrations of 0.01 mg/mL, 0.03 mg/mL, and 0.1 mg/mL and injected intraperitoneally with a volume of 10 mL/Kg 30 min before and after the operation.

### Experimental Animals

Specific-pathogen-free (SPF) adult Sprague–Dawley rats were purchased from the Hunan Slake Jingda Company (licence number: SCXK (Xiang) 2019–0004). The animals were housed in groups for breeding, with one male and two females per cage. Seven-day-old male and female rats weighing 12–18 g were selected for the experiment. Neonatal rats were housed with their mothers at a temperature of 20–25 °C and a relative humidity of 50–60% on a 12-h/12-h light/dark cycle. All animal experiments were approved by the Biomedical Research Ethics Committee of Gannan Medical University.

### Reagents

Tris, glycine, 30% acrylamide, sodium dodecyl sulfate (SDS), Tween-20, PMSF, and high-efficiency RIPA tissue/cell lysis buffer were purchased from Solarbio Science & Technology Company (Beijing, China). Anti-cathepsin Z (CTSZ; #ab182575), anti-C3 (#ab200999), anti-CD11b (#ab133357), anti-CD16 (#ab211151), and anti-NeuN (#ab104224) antibodies were purchased from Shanghai Abcam Company. Anti-GBP2 antibody (#11,854–1-AP) was purchased from Proteintech. Anti-GFAP (#MA5-12,023), anti-β-actin (#MA5-15,739), anti-β-tubulin (#MA 5–11,732), Alexa Fluor 555-conjugated goat anti-mouse IgG (#a21424), and Alexa Fluor 488-conjugated goat anti-rabbit IgG (#a11034) antibodies were purchased from Invitrogen. Enhanced chemiluminescence (ECL) Western blot detection reagent (#32,106) was purchased from Thermo Fisher Scientific, and 2,3,5-triphenyltetrazolium chloride (TTC, #T8877-25G) was purchased from Sigma. A Fluoro-Jade C Kit (FJC, TR-100-FJT) was purchased from Biosensis.

### Establishment of a Neonatal Rat Model of HIE and Drug Treatment

The Rice-Vannucci HIE model was constructed using neonatal SD rats [23]. At postnatal day 7, SD rats were anaesthetized with 2–3% isoflurane (the time from induction to awakening did not exceed 10 min) and fixed in a supine position. A median neck incision was made with the use of a microscope. The right common carotid artery was separated and electrocoagulated, and then, the incision was sutured. After the neonatal rats recovered from anaesthesia, they were returned to their mothers for feeding. Ninety minutes after the operation, the neonatal rats were placed in an anoxic tank at 37 °C for 1 h (7% O_2_) and then returned to normal oxygen conditions and to their mothers for feeding. In the GSS treatment group, the right common carotid artery was separated and electrocoagulated, and the drug was administered intraperitoneally 30 min before the operation and after reperfusion. In the hypoxia–ischaemia (HI) group, the right common carotid artery was isolated and electrocoagulated, and the same volume of normal saline was injected intraperitoneally. In the sham group, the right common carotid artery was isolated but not electrocoagulated, and the same volume of normal saline was injected intraperitoneally.

### Neurological Assessment

Behavioural evaluations were performed in the acute phase (24 h after the operation) to assess the neurological deficits of neonatal rats in each group [24]. To test the righting reflex, each newborn rat was placed face up on a flat board, with its head, neck, and back touching the board, and the time it took the rat to turn over from the supine position and stand on all four limbs was recorded. In the cliff avoidance test, each newborn rat was placed with two-thirds of its body on the edge of a platform, and the latency of the rat to retreat or move sideways away from the edge of the platform was recorded. In the negative geotaxis experiment, each newborn rat was placed on a rough board at a 45-degree angle from the horizontal plane, close to the upper end of the board to prevent it from seeing or touching objects on the table and escaping. The rat was initially oriented with its head facing downhill; the latency of the animal to turn its head and begin to climb uphill was recorded. In the hanging test, a string with a diameter of 2 mm was suspended between two points, and each newborn rat was allowed to grip the string with its forepaws. The rat was released to hang by its paws, and its latency to fall was recorded. All experiments were repeated three times for each rat, and the average value was taken.

### TTC Staining

After 24 h of ischaemia-hypoxia, the cerebral infarct area was measured by TTC staining. After anaesthesia with ether, the rats were decapitated, and their brains were removed and frozen in a − 20 °C freezer for 30 min. The brains were cut into four consecutive coronal slices (2 mm thick), placed in 0.5% TTC solution, and incubated in a water bath in the dark for 30 min at 37 °C. During this period, the brain slices were turned every 5 min. After staining, the brain slices were washed twice with PBS, fixed with 4% paraformaldehyde, observed, and photographed. The cerebral infarct area was analysed with ImageJ software and calculated as follows: percentage of cerebral infarct area (%) = (area of normal brain tissue on the side opposite the infarct + area of the infarct − area of the infarcted side) / area of normal brain tissue on the side opposite the infarct × 100%.

### Preparation of Brain Tissue Samples

Neonatal rats were anaesthetized with 2–3% isoflurane and perfused with normal saline and 4% paraformaldehyde. The rats were decapitated, and the brains were removed, fixed in 4% paraformaldehyde for 24 h, and then immersed in 20% and 30% sucrose solution until they sank. After being embedded in OCT compound, the brains were cut into 30-μm consecutive coronal slices using a freezing microtome.

### RNA-Seq

Total RNA was extracted from brain tissues with a TRIzol kit, and RNA integrity was assessed by using agarose gel electrophoresis and an Agilent 2100 Bioanalyzer. mRNA with a poly(A) tail was enriched by oligo(dT) magnetic beads, and the NEBNext® Ultra™ RNA Library Prep Kit for Illumina was used to build a library. Then, Illumina sequencing was performed. To ensure the quality and reliability of the data, raw sequencing reads were first trimmed with the Fastp programme to remove adaptor sequences, short sequences, low-quality bases, and reads with poly-Ns [25]. The clean reads used in the subsequent analysis were obtained after filtering of the original data, inspection of the sequencing error rate, and inspection of GC content distribution. The clean reads were mapped to the rat genome (*Rattus norvegicus* 6.0, Rnor 6.0) with HISAT2 (version 2.2.1) [26].

### Bioinformatics Analysis

The differentially expressed genes between the sham and HI groups and between the HI and GSS groups were identified by using the DESeq2 R package [27]. DESeq2 utilises negative binomial generalised linear models to test for differential expression. Genes discovered with a *p*-adjusted value less than 0.05 and |log fold change (FC)|> 0 were considered significantly differentially expressed. Gene Ontology (GO) enrichment was performed by the R package clusterProfiler (v4.2.2) to identify biological processes (BP), molecular functions (MF), and cellular components (CC) associated with the significantly differentially expressed genes. Kyoto Encyclopedia of Genes and Genomes (KEGG) pathway analyses were performed to analyse differentially expressed genes with the R package clusterProfiler (v4.2.2) [28], and the first 10 annotations with significant enrichment were selected for visualisation.

### Western Blots

Right cerebral cortex tissues were extracted from the rats 24 h after the operation for Western blot analysis. RIPA lysis buffer containing phosphatase inhibitor was added to the brain tissues. The tissues were homogenised on ice for 1 h and then centrifuged at 4 °C and 12,000 r/min for 10 min, and the supernatant was collected. The protein concentration was determined with a BSA protein quantification kit according to the manufacturer’s instructions. Equal amounts of protein were loaded on an SDS‒PAGE gel for electrophoresis and then transferred onto a PVDF membrane. The membrane was blocked with 5% skim milk at room temperature for 1 h and then incubated overnight with the following primary antibodies: anti-C3 (1:2000), anti-CTSZ (1:1000), anti-GFAP (1:1000), anti-GBP2 (1:1000), anti-CD11b (1:1000), anti-SLC17A7 (1:2000), anti-CD16 (1:1000), and anti-β-actin (1:1000). The next day, the membrane was washed with TBST 3 times for 10 min each, incubated with the corresponding secondary antibody (1:5000) at room temperature for 1 h, and then washed with TBST 3 times for 10 min each. Finally, the membrane was incubated with ECL reagent for 1 min, and a chemiluminescence imaging system (Amersham™ Imager 600, USA) was used to capture the protein bands. The grey values of the protein bands were calculated with ImageJ software.

### Fluoro-Jade C Staining

Degenerating neurons after HIE were visualised using a Fluoro-Jade C staining kit according to the manufacturer’s instructions. The brain slice was incubated in a mixed solution containing 9 parts 80% ethanol and 1 part sodium hydroxide for 5 min, in a solution including 9 parts distilled water and 1 part potassium permanganate for 10 min, and then in a solution composed of 8 parts distilled water, 1 part Fluoro-Jade C, and 1 part DAPI for 10 min in the dark. Images were obtained with a laser confocal microscope, and at least 3 fields of view were selected for each slice. The number and fluorescence intensity of Fluoro-Jade C–positive cells in each field of view were calculated with ImageJ software. In this experiment, 3 rats in each group were observed, and 3 slices were selected from each animal.

### Immunofluorescence

The prepared slices were washed with PBST 3 times for 10 min each and blocked with 3% BSA at room temperature for 1 h. Subsequently, the brain slices were incubated with anti-NeuN primary antibody at 4 °C overnight, washed with PBST 3 times for 10 min each, incubated with the corresponding secondary antibody at room temperature for 1 h, and then washed 3 times for 10 min each. The brain slices were mounted on glass slides, dried, and sealed with an anti-fluorescence quenching agent containing DAPI. At least 3 images per slice were obtained with a laser confocal microscope, and the fluorescence intensity and percentage of positive cells were analysed with ImageJ software. In this experiment, 3 rats in each group were observed, and 3 slices were selected from each animal.

### Statistical Analysis

GraphPad Prism 8 software was used for statistical analysis. The data are expressed as the mean ± SD. One-way ANOVA was used to compare the differences between groups, and Dunnett’s test was used for multiple pairwise comparisons between groups. *p* < 0.05 was considered significant.

## Results

### GSS Treatment Alleviates Brain Damage and Improves Neurological Function in Neonatal Rats with HIE

**Fig. 1:**
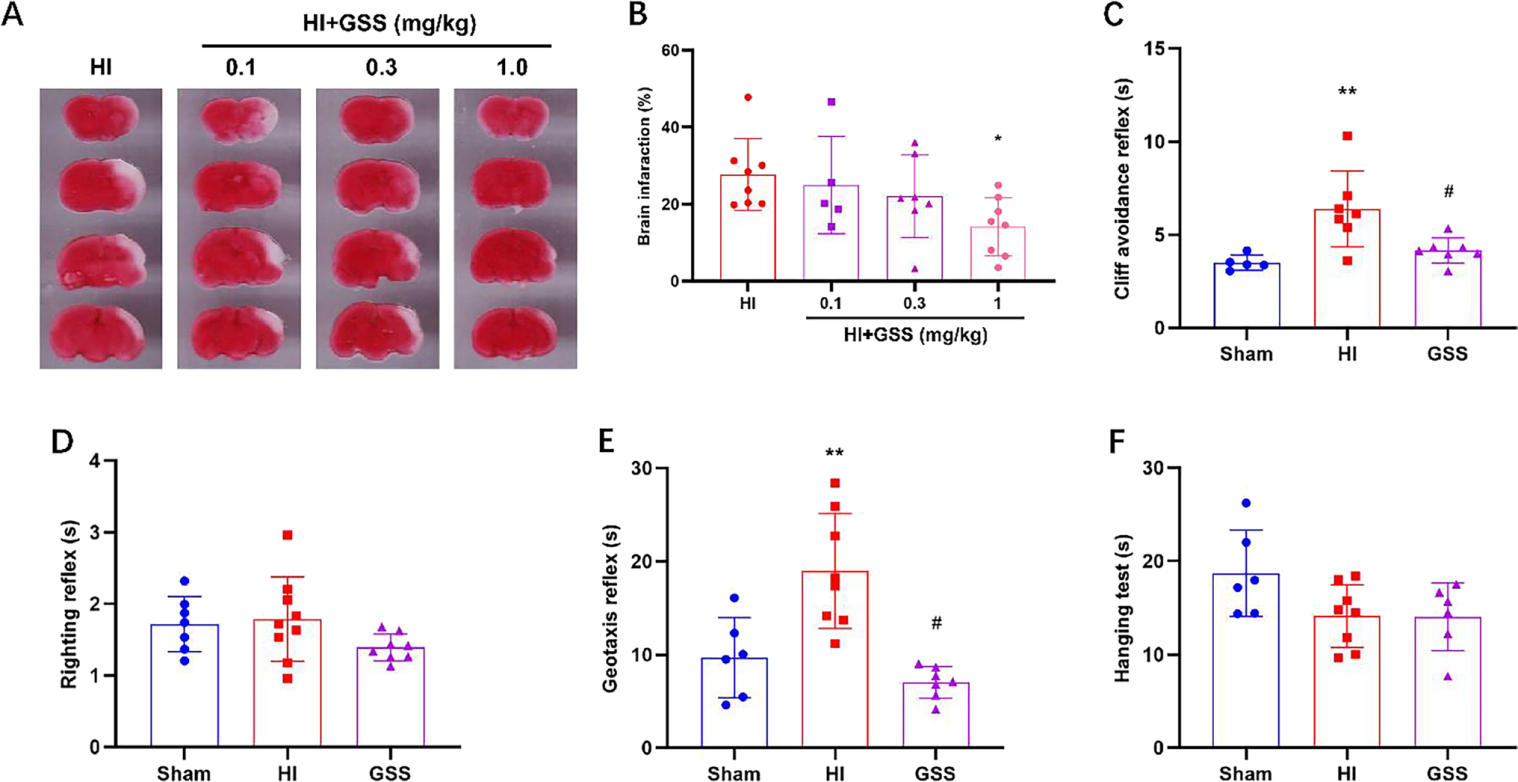
GSS treatment alleviates brain damage and improves neurological function in neonatal rats with HIE. **A**, **B** The cerebral infarct volume after HI in brain tissue in the different groups (vehicle, 0.1, 0.3, 1.0 mg/kg GSS) was analysed by TTC staining. **C** The righting reflex. **D** The cliff avoidance reflex. **E** The negative geotaxis reflex. **F** The hanging test. ^**^p < 0.01 vs. sham; ^#^*p* < 0.05 vs. vehicle

To explore the optimal dose of GSS for neuroprotection against HIE, we initially tested three doses: 0.1 mg/kg, 0.3 mg/kg, and 1 mg/kg. TTC staining results showed that the cerebral infarct area was significantly reduced in the 1 mg/kg GSS group compared with the HI group (Fig. 1A, B). Thus, this dose was used in the following experiments.

Neurological function was assessed 24 h after the operation. There were no significant differences in performance on the righting reflex test or the hanging test (Fig. 1C, F). In the cliff avoidance and geotaxis tests, the latency of the HI group was significantly increased compared with that of the sham group, and the latency of the GSS group was significantly reduced compared with that of the sham group (Fig. 1D, E). These results indicate that GSS has significant neuroprotective effects in neonatal rats with HIE.

### GSS Treatment Inhibits Neuronal Loss and Degeneration in Neonatal Rats with HIE

**Fig. 2:**
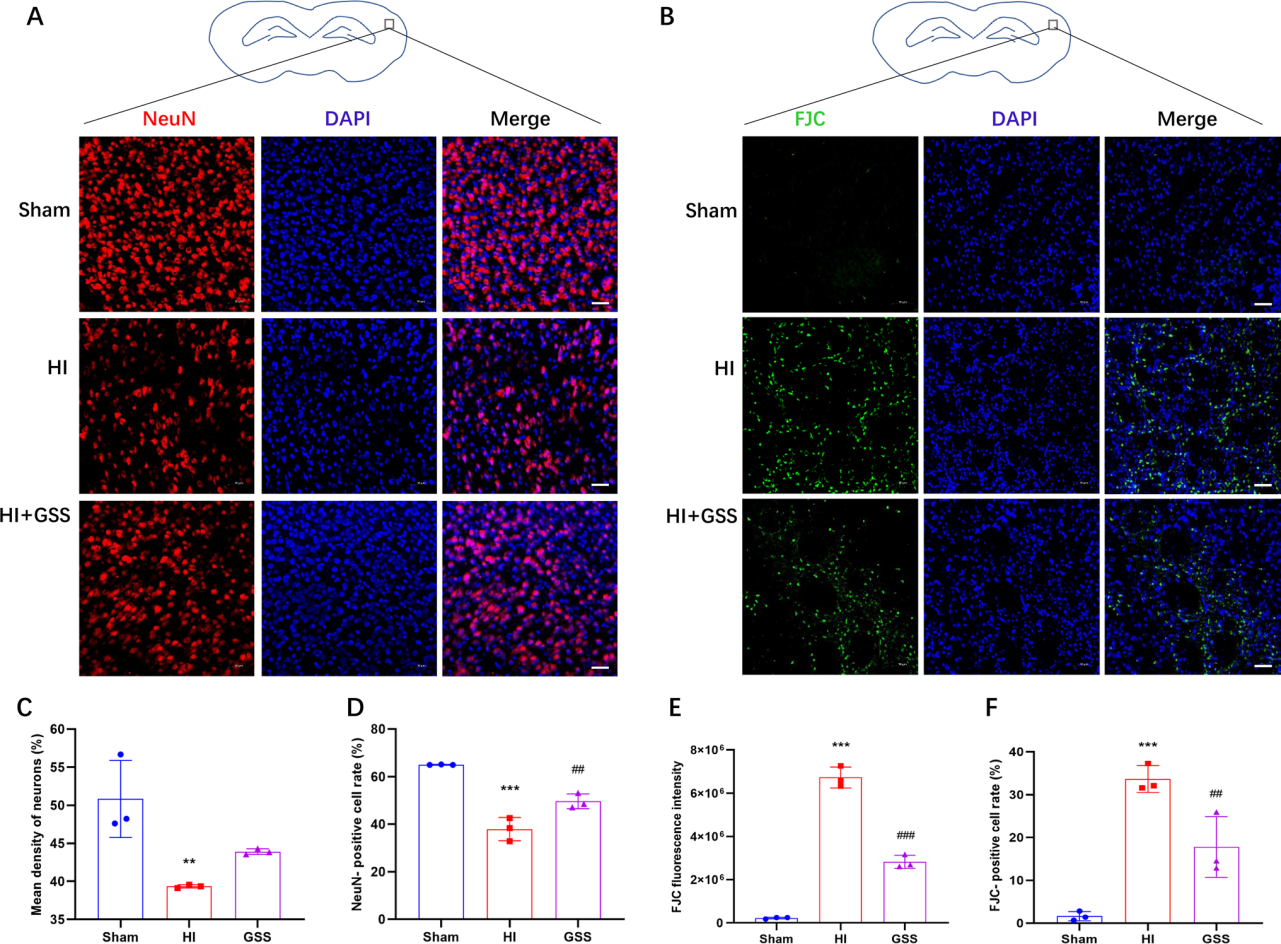
GSS treatment inhibits neuronal loss and degeneration in neonatal rats with HIE. **A**, **B** Representative image of NeuN and FJC immunofluorescence (scale bar = 50 μm, *n* = 3). **C**, **D** NeuN average fluorescence intensity and positive cells rate. **E**, **F** FJC average fluorescence intensity and positive cells rate. ^**^p < 0.01 and ^***^p < 0.001 vs. sham; ^##^*p* < 0.01 and ^###^*p* < 0.001 vs. vehicle

Next, we further explored the protective effect of GSS treatment on neurons in neonatal rats with HIE. NeuN immunofluorescence and Fluoro-Jade C staining were used to assess neuronal loss and degeneration in the cerebral cortices of neonatal rats after ischaemia-hypoxia (Fig. 2A, B). NeuN immunofluorescence results showed that, compared with the sham group, the HI group exhibited significant reductions in the number of NeuN-positive cells and NeuN fluorescence intensity as well as severe neuronal loss, whilst GSS treatment significantly inhibited neuronal loss in the HI group (Fig. 2C, D). The Fluoro-Jade C staining results showed that, compared with that in the sham group, the number of Fluoro-Jade C–positive cells and the presence of neurodegeneration in the HI group were significantly increased, whilst GSS treatment significantly inhibited neurodegeneration (Fig. 2E, F). In short, GSS has a significant protective effect on neurons.

### Identification of DEGs

**Fig. 3:**
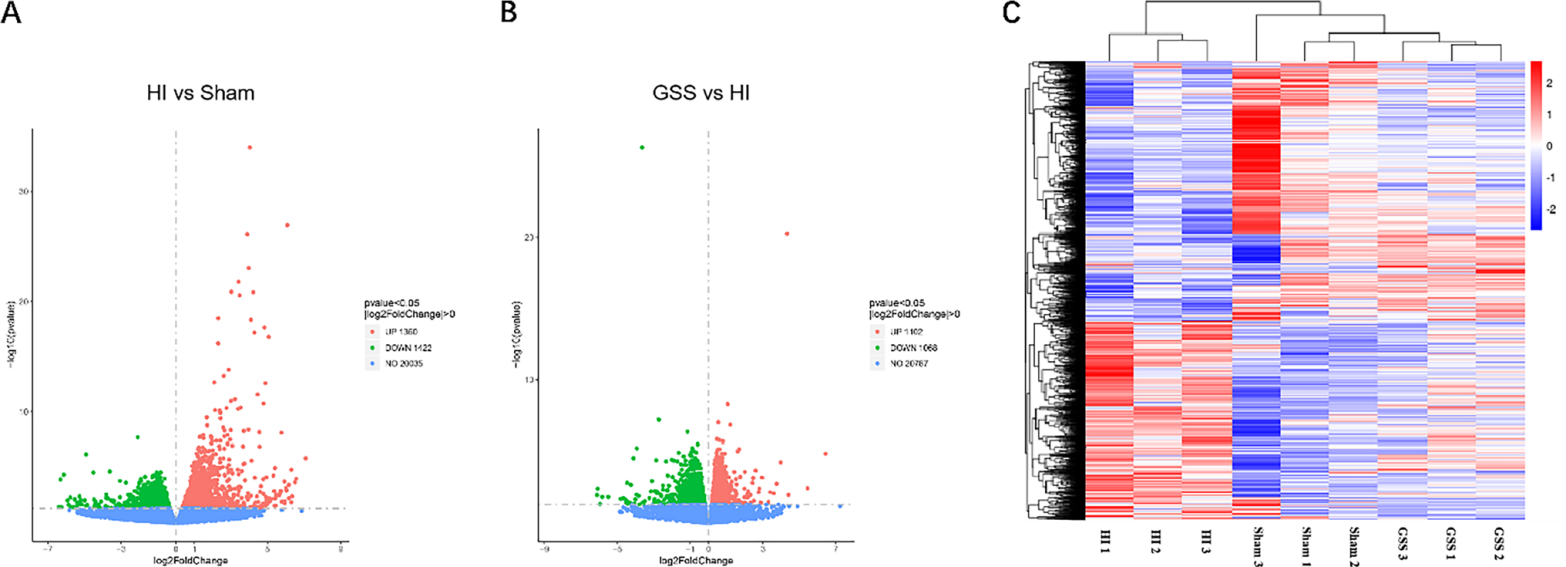
Identification of DEGs. **A** and **B** are the volcano maps of HI vs. sham and GSS vs. HI combination respectively. Red dots represent upregulated genes, green dots represent downregulated genes, and blue dots represent genes with no significant difference. **C** Cluster heat map of DEGs. DEGs standard: *p* < 0.05, | log2 fold change |> 0

We used RNA-Seq to determine the gene expression profiles in cortical tissues from the sham, HI, and GSS groups. A total of 2782 DEGs, of which 1360 were upregulated and 1422 were downregulated, were identified in the HI group compared with the sham group (Fig. 3A). A total of 2170 DEGs, of which 1102 were upregulated and 1068 were downregulated, were identified in the GSS group compared with the HI group (Fig. 3B). Cluster analysis revealed that the gene expression patterns in the sham group, HI group, and GSS group were significantly different and that the pattern in the GSS group was similar to that in the sham group (Fig. 3C).

### GO and KEGG Functional Enrichment Analysis of Genes Upregulated and Downregulated by GSS

**Fig. 4:**
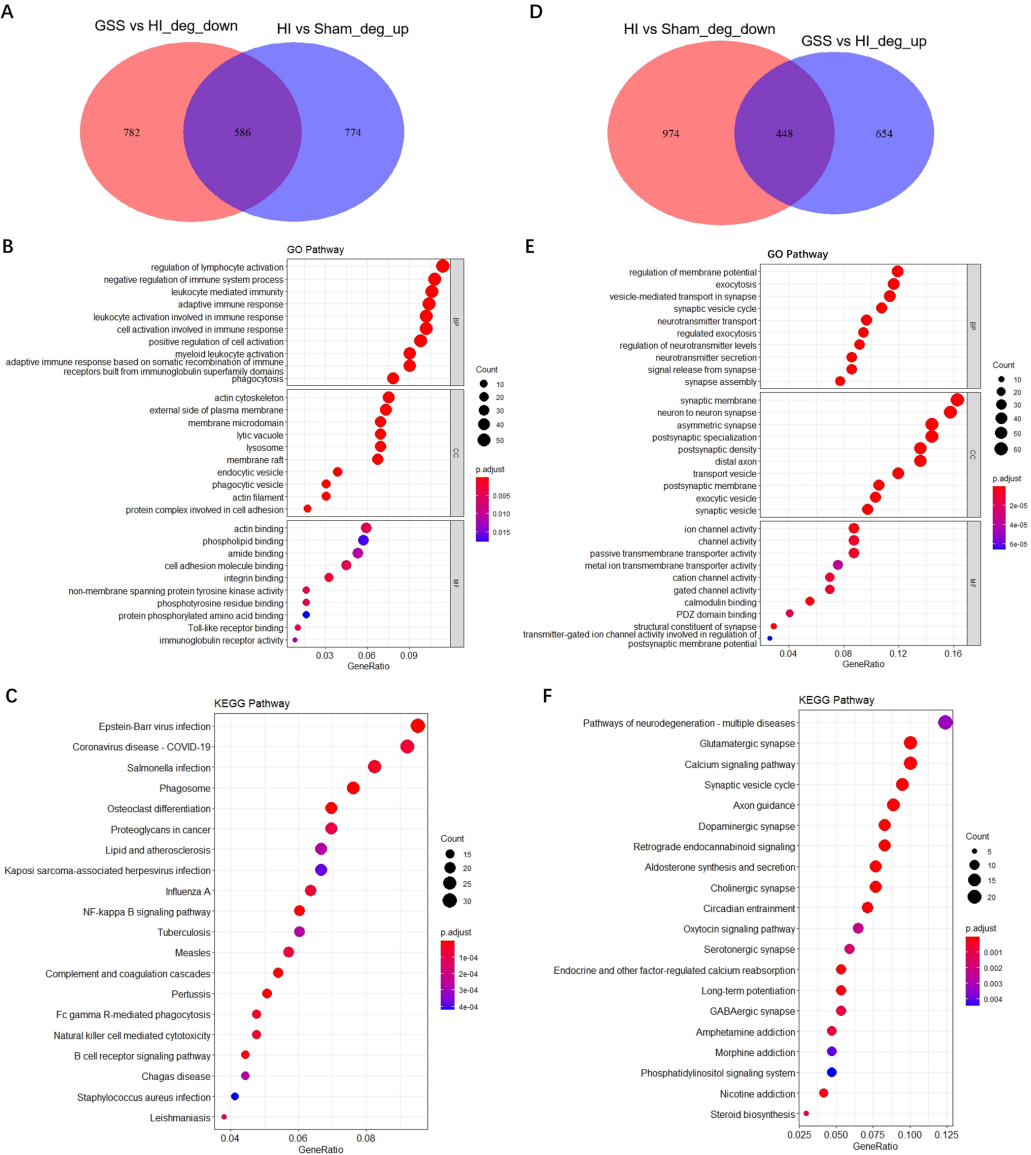
GO and KEGG functional enrichment analysis of genes upregulated and downregulated by GSS. **A** Differential gene Venn diagram of HI vs. Sham_up and GSS vs. HI_down. **B**, **C** GO and KEGG enrichment analysis bubble chart of DEGs downregulated by GSS. **D** Differential gene Venn diagram of HI vs. Sham_down and GSS vs. HI_up. **E**, **F** GO and KEGG enrichment analysis bubble chart of DEGs upregulated by GSS. DEGs standard: *p* < 0.05, |log2 fold change|> 0

To further investigate the molecular mechanism underlying the protective effect of GSS against HIE-induced brain injury in neonatal rats, we identified 586 DEGs that overlapped between HI vs. Sham_up and GSS vs. HI_down and 448 DEGs that overlapped between HI vs. Sham_down and GSS vs. HI_up (Fig. 4A, D). This indicates that GSS treatment downregulated the expression of 586 DEGs and upregulated the expression of 448 DEGs. Next, clusterProfiler software was used to perform GO and KEGG functional enrichment analyses of the upregulated and downregulated DEGs.

GO enrichment analysis showed that a total of 1087 and 603 GO terms were significantly enriched for the 594 downregulated DEGs and 471 upregulated DEGs, respectively. Downregulated DEGs participated in “regulation of lymphocyte activation”, “adaptive immune response”, “phagocytosis”, “lysosome”, “phospholipid binding”, “cell adhesion molecule binding”, “Toll-like receptor binding”, and other pathways (Fig. 4B). Upregulated DEGs participated in “regulation of neurotransmitter levels”, “exocytosis”, “synaptic vesicle cycle”, “synaptic membrane”, “neuron to neuron synapse”, “ion channel activity”, and other pathways (Fig. 4E).

KEGG enrichment analysis showed that a total of 60 pathways and 33 pathways were enriched for the 594 downregulated DEGs and 471 upregulated DEGs, respectively. Downregulated DEGs were significantly enriched in the pathways “Phagosome”, “NF-kB signalling pathway”, “Complement and coagulation cascades”, “Fc gamma R-mediated phagocytosis”, “Natural killer cell mediated cytotoxicity”, and “B cell receptor signalling pathway” (Fig. 4C). Upregulated DEGs were significantly enriched in “Pathways of neurodegeneration—multiple diseases”, “Glutamatergic synapse”, “Calcium signalling pathway”, “Synaptic vesicle cycle”, “Dopaminergic synapse”, “Aldosterone synthesis and secretion”, “Cholinergic synapse”, “Long-term potentiation”, and others (Fig. 4F). These results indicate that GSS intervenes in the process of HIE-induced brain injury by participating in multiple pathways.

### Validation of the RNA-Seq Results by Western Blotting

**Fig. 5:**
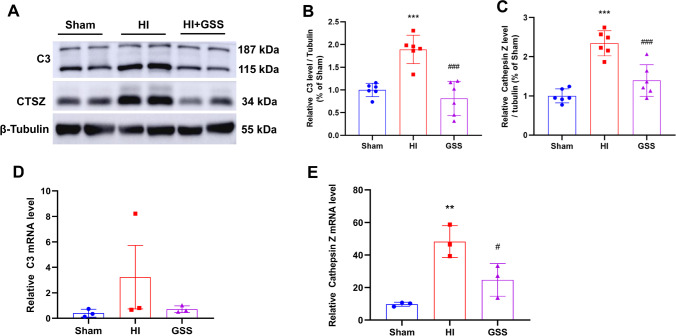
Verification of the RNA-Seq results of C3 and CTSZ by Western blotting. **A** Western blot band of C3 and CTSZ. **B**, **C** Statistical analysis of C3 and CTSZ protein expression. **D**, **E** Relative mRNA level of C3 and CTSZ evaluated by RNA-Seq. ^**^p < 0.01 and ^***^p < 0.001 vs. sham; ^#^*p* < 0.05 and ^###^*p* < 0.001 vs. vehicle

Finally, the DEGs identified by RNA-Seq were validated by Western blotting. We selected 7 genes for validation. The Western blotting results were consistent with the RNA-Seq data. Compared with the sham group, the HI group had significantly increased expression of complement C3, indicating that complement pathway components accumulate in the HI group and that GSS treatment can significantly reduce the expression of complement C3 (Fig. 5A, B), which is consistent with the RNA-Seq results (Fig. 5E). Cathepsin Z (CTSZ) is a member of the cysteine cathepsin family, is a lysosomal cysteine proteinase, and has exopeptidase activity [29]. The results showed that CTSZ expression was increased after HIE and that GSS treatment inhibited the increased expression of this protein (Fig. 5A, C).

**Fig. 6:**
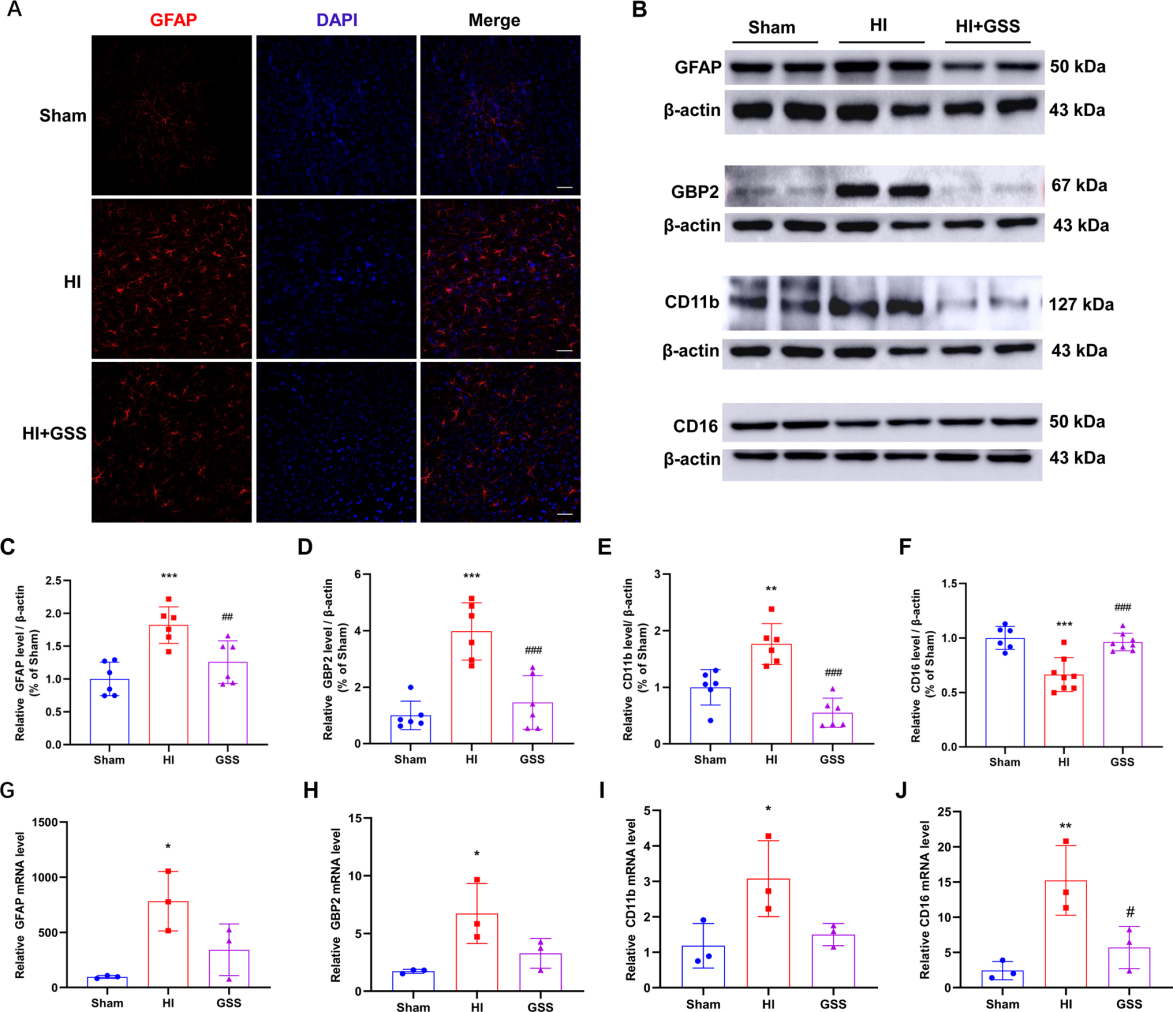
Verification of the RNA-Seq results of GFAP, GBP2, CD11b and CD16 by Western blotting and immunofluorescence. **A** Representative image of GFAP immunofluorescence (scale bar = 50 μm, *n* = 3). **B** Western blot band of GFAP, GBP2, CD11b, and CD16. **C**–**F** Statistical analysis of GFAP, GBP2, CD11b, and CD16 protein expression. **G**–**J** Relative mRNA level of GFAP, GBP2, CD11b, and CD16 evaluated by RNA-Seq. ^*^p < 0.05, ^**^p < 0.01 and ^***^p < 0.001 vs. sham; ^#^*p* < 0.05, ^##^*p* < 0.01, and ^###^*p* < 0.001 vs. vehicle

**Fig. 7:**
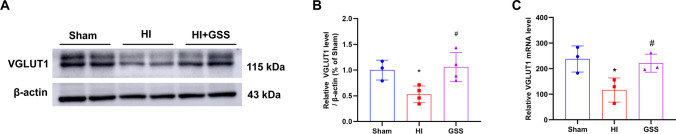
Verification of the RNA-Seq result of VGLUT1 by Western blotting. **A** Western blot band of VGLUT1. **B** Statistical analysis of VGLUT1 protein expression. **C** Relative mRNA level of VGLUT1 evaluated by RNA-Seq.^*^p < 0.05 vs. sham; ^#^*p* < 0.05 vs. vehicle

In addition, we found that the expression of the activation markers GFAP and CD11b in astrocytes and microglia in the HI group was significantly increased. The increased expression of GBP2 indicated that astrocytes were polarised towards the A1 phenotype in the HI group. However, GSS treatment reduced the expression of GFAP, GBP2, and CD11b (Fig. 6A–E), which is consistent with the RNA-Seq results (Fig. 6G–I). The results of Western blot analysis revealed that CD16 expression was opposite from the RNA-Seq results (Fig. 6F, J). The expression of vesicular glutamate transporter (VGLUT1) was increased in the HI group, but GSS treatment reduced its expression, which is consistent with the RNA-Seq results (Fig. 7A–C).

## Discussion

Brain damage caused by neonatal HIE is a continuous process that involves three stages. In the acute phase, cerebral blood flow decreases, and oxygen, glucose, and ATP levels decrease sharply, inducing energy failure, cytotoxic oedema, Ca^2+^ accumulation, excitatory glutamate release, and neuronal death [30]. This phase lasts for approximately 6 h, after which secondary energy failure occurs and the typical symptoms, such as acute inflammation, oxidative stress, and seizures, are further exacerbated [31]. The third stage occurs within a few months after acute injury and involves brain remodelling, astrocyte degeneration, and late cell death [32]. Currently, TH is the only intervention approved for the treatment of neonatal HIE. However, the treatment window for TH is narrow (within 6 h after birth), and its efficacy is not entirely satisfactory [33]. Thus, alternative treatments are urgently needed.

In this study, we found that GSS also showed a strong neuroprotective effect in an HIE model. The protective effect of GSS against HIE was dose-dependent, with the best effect at 1 mg/kg GSS; we selected this dose for use in subsequent experiments. In addition, we found that at this dose, GSS not only alleviated brain damage and neurological deficits in neonatal rats with HIE but also reduced neuronal loss and degeneration. Finally, we used RNA-Seq to reveal that GSS protects against HIE through multiple genes, proteins, and pathways.

Increasing evidence suggests that neuronal apoptosis is one of the main pathological processes of neurological impairment after HIE [34]. In addition to the early necrosis of cells in the ischaemic core, the cell death caused by cerebral ischaemia and hypoxia also includes the delayed death of susceptible neurons in the peripheral area [35]. Our results demonstrated that GSS treatment significantly increased the number of NeuN-positive neurons and Fluoro-Jade C–positive neurons, suggesting that GSS treatment can promote neurological recovery in neonatal rats and reduce neuronal loss and degeneration caused by cerebral ischaemia and hypoxia.

To further study the molecular mechanism of the neuroprotective effect of GSS on HIE neonatal rats, we screened the DEGs upregulated and downregulated by GSS using RNA-Seq and performed GO and KEGG enrichment analyses on the DEGs. GO and KEGG enrichment analyses revealed that GSS is mainly involved in pathways such as “Phagosome”, “NF-kB signalling pathway”, “Complement and coagulation cascades”, “Fc gamma R-mediated phagocytosis”, “Long-term potentiation”, “Pathways of neurodegeneration-multiple diseases”, and “Glutamatergic synapse”, indicating that GSS intervened in the process of HI injury in neonatal rats by participating in multiple pathways related to inflammation. Previous studies have shown that GSS can ameliorate cerebral ischaemic injury in tMCAO rats by inhibiting neuroinflammation [14, 15]. These results indicated that GSS can interfere with several inflammation-related pathways and exert significant protective effects on HIE neonatal rats. Next, we selected 7 DEGs with significantly different *p* values and |log2 FC| values in these pathways for validation. Six genes were involved in the inflammatory response to brain injury in HIE neonatal rats, including C3, CTSZ, GFAP, GBP2, CD11b, and CD16. In addition, VGLUT1 is involved in glutamate toxicity injury in HIE neonatal rats.

The complement system is an important part of innate immunity and is activated through the classical pathway, lectin pathway, and alternative pathway. Studies have shown that the inflammatory fragments generated by the overactivation of the complement system are one of the important causes of inflammatory response impairment in the brain [36], in which complement C3 plays an important role. Studies have found that complement C3 accumulates in large quantities in neurons, aggravating HIE-induced brain injury [37]. This finding was also verified by our results. In mice with multiple sclerosis, a lack of CTSZ can significantly reduce the level of IL-1β in the serum and reduce neuroinflammation. This is the first study to prove that the expression of CTSZ is related to neuroinflammation [38]. Our results also suggest that GSS may reduce the inflammatory response by decreasing the expression of CTSZ, but more experiments are still needed.

Astrocytes are the most abundant glial cells in the central nervous system (CNS) and are involved in cerebral blood flow regulation, myelination, neurotransmission, and glutamate metabolism [39–41]. Microglia, as CNS macrophages, are the main immune cells in the nervous system and play roles in neuronal regulation, synaptic pruning, and axonal guidance [42–44]. When the CNS is damaged, both astrocytes and microglia are activated. Activated microglia are polarised towards the proinflammatory M1 phenotype or the anti-inflammatory M2 phenotype, and activated astrocytes are polarised towards the proinflammatory A1 phenotype or the anti-inflammatory A2 phenotype [45, 46]. Altering the phenotypes of astrocytes and microglia has become a new therapeutic strategy [47, 48]. In this study, GSS was found to decrease the high expression of GFAP and GBP2 after HIE in neonatal rats, indicating depressive effects of activation and A1-type polarisation on astrocytes. CD16 is one of the markers of microglial polarisation into the M1 type after activation [49, 50]. GSS also reduced the transcription of both CD11b and CD16 mRNA as well as the protein level of CD11b, showing an inhibitory effect on the activation and polarisation of microglia. However, the expression of the M1 marker CD16 at the gene and protein levels was inconsistent, which is thought to be the reason for the asynchrony of RNA transcription and protein translation.

Glutamate is the main excitatory neurotransmitter in the CNS [51]. VGLUT1, which is mainly located on the membranes of presynaptic glutamate vesicles, loads glutamate into synaptic vesicles, participates in glutamate metabolism, and is a specific biomarker of glutamatergic neurons and glutamatergic synapses [52]. Studies have shown that VGLUT1 expression determines the filling rate of excitatory synaptic vesicles and directly affects glutamate receptors and synaptic transmission [53]. Therefore, reduced VGLUT expression can lead to abnormal levels of the excitatory neurotransmitter glutamate, which is involved in the progression of CNS diseases such as learning and memory disorders, PD, AD, depression, and schizophrenia [54–58]. In this study, GSS reduced the expression of VGLUT1 after cerebral ischaemia and hypoxia in neonatal rats, suggesting a new mechanism by which GSS alleviates glutamate toxicity in HIE.

Our previous study showed that GSS alleviated cerebral ischaemia/reperfusion-induced neuroinflammation in adult rats by inhibiting the NF-κB signalling pathway [14]. In the current study, the results of RNA-Seq analysis found that GSS inhibits NF-κB signalling and reduces the activation and polarisation of microglia and astrocytes by inhibiting GFAP, GBP2, CD11b, and CD16, thereby inhibiting neuroinflammation. It has also been reported that activation of G protein–coupled oestrogen receptor (GPER), an oestrogen membrane receptor, can suppress neuroinflammation by inhibiting NF-κB signalling and inhibit excitotoxicity by activating ERK signalling. Based on the above evidence, it can be speculated that GSS, a phytoestrogen, inhibits neuroinflammation and excitotoxic injury, possibly by activating GPER.

In summary, we used RNA-Seq to identify the genes and functional pathways involved in the protective effect of GSS on the brains of neonatal rats with HIE. Although we validated the results of RNA-Seq using Western blotting, more experiments are needed to further explore the identified DEGs and pathways. In conclusion, this study identifies many of the potential therapeutic targets of GSS in the treatment of HIE-induced brain injury.

## Data Availability

The datasets used during the current study are available from the corresponding author on reasonable request.

## Abbreviations

CTSZ: Cathepsin Z
ECL: Enhanced chemiluminescence
FJC: Fluoro-Jade C
Gen: Genistein
GSS: Genistein-3′-sodium sulfonate
HIE: Hypoxic-ischaemic encephalopathy
SD: Sprague–Dawley
SDS: Sodium dodecyl sulfate
TH: Therapeutic hypothermia
tMCAO: Transient middle cerebral artery occlusion
TTC: 2,3,5-Triphenyltetrazolium chloride
VGLUT1: Vesicular glutamate transporter
